# 15 Years of Microstate Research in Schizophrenia – Where Are We? A Meta-Analysis

**DOI:** 10.3389/fpsyt.2016.00022

**Published:** 2016-02-26

**Authors:** Kathryn Rieger, Laura Diaz Hernandez, Anja Baenninger, Thomas Koenig

**Affiliations:** ^1^Translational Research Center, University Hospital of Psychiatry, University of Bern, Bern, Switzerland; ^2^Center for Cognition, Learning and Memory, University of Bern, Bern, Switzerland

**Keywords:** microstates, schizophrenia, EEG, neurofeedback, saliency

## Abstract

Schizophrenia patients show abnormalities in a broad range of task demands. Therefore, an explanation common to all these abnormalities has to be sought independently of any particular task, ideally in the brain dynamics before a task takes place or during resting state. For the neurobiological investigation of such baseline states, EEG microstate analysis is particularly well suited, because it identifies subsecond global states of stable connectivity patterns directly related to the recruitment of different types of information processing modes (e.g., integration of top-down and bottom-up information). Meanwhile, there is an accumulation of evidence that particular microstate networks are selectively affected in schizophrenia. To obtain an overall estimate of the effect size of these microstate abnormalities, we present a systematic meta-analysis over all studies available to date relating EEG microstates to schizophrenia. Results showed medium size effects for two classes of microstates, namely, a class labeled C that was found to be more frequent in schizophrenia and a class labeled D that was found to be shortened. These abnormalities may correspond to core symptoms of schizophrenia, e.g., insufficient reality testing and self-monitoring as during auditory verbal hallucinations. As interventional studies have shown that these microstate features may be systematically affected using antipsychotic drugs or neurofeedback interventions, these findings may help introducing novel diagnostic and treatment options.

## Introduction

Schizophrenia is a psychiatric disorder showing a broad range of deficits across a multitude of task demands (1). Therefore, an explanation common to all these abnormalities has to be sought independently of any particular task but instead during baseline states, i.e., before a task takes place or during rest. In addition, and under the scope of the “disconnection hypothesis” of schizophrenia (2), it does not seem reasonable to assume that an isolated local system can account for these deficits, but rather, a dysfunctional integration among neural systems. Research on the neurobiology of schizophrenia accumulates a large body of evidence supporting this approach (3–5). Relevant findings among others include abnormal pruning of connections during adolescence (6), structural abnormalities in white matter tracks (7, 8) and alterations of brain functional connectivity during task execution as well as during rest (9, 10).

EEG research has long reported abnormalities in schizophrenia patients [see Ref. (11) for an overview] and has provided substantial support to the disconnection hypothesis (12–14). Importantly, evidence for abnormal dynamics of particular transiently stable functional brain networks has repeatedly been reported in relation to schizophrenia. The aforementioned functional networks were identified using EEG microstate analysis (15, 16) and are the so-called EEG resting-state networks (15–18). For a comprehensive outlook on microstates, we refer the reader to the recent review by Khanna et al. (18).

Microstate analysis of the ongoing EEG shows subsecond periods of quasi-stable spatial configurations that have been linked to fMRI resting-state networks (19). The fact that the simultaneity of events across distributed regions is a defining property of microstates coincides well with theoretical considerations about the role of synchronization for the integration of brain activity into something that is subjectively experienced as unitary (20). The sequence of microstates and the rules potentially governing these sequences [the so-called microstate syntax (21)] may represent the subsecond switching between various types of such integrative states (18).

Interestingly, the observed microstate configurations repeat within and across subjects. This allows the investigation of a limited set of prototypical microstate configurations using spatial clustering algorithms (16, 22). These prototypical configurations can then be used to efficiently quantify multi-subject EEG resting-state data. Over the past 15 years, ongoing research has been able to systematically link changes in EEG microstate quantifiers in domains, such as schizophrenia research (13, 21, 23–28), development (29), perceptual modes (30), and fMRI resting-state networks (19) or sleep (31).

In this paper, we present the results of a meta-analysis on all publications that so far have bound together schizophrenia and microstates. As interventional studies have shown that these microstate features may be systematically affected using conventional [i.e., medication as in (13)] or neurofeedback (NFB) interventions (32), the findings may help boosting the development of novel diagnostic and treatment options.

## Materials and Methods

**Figure 1:**
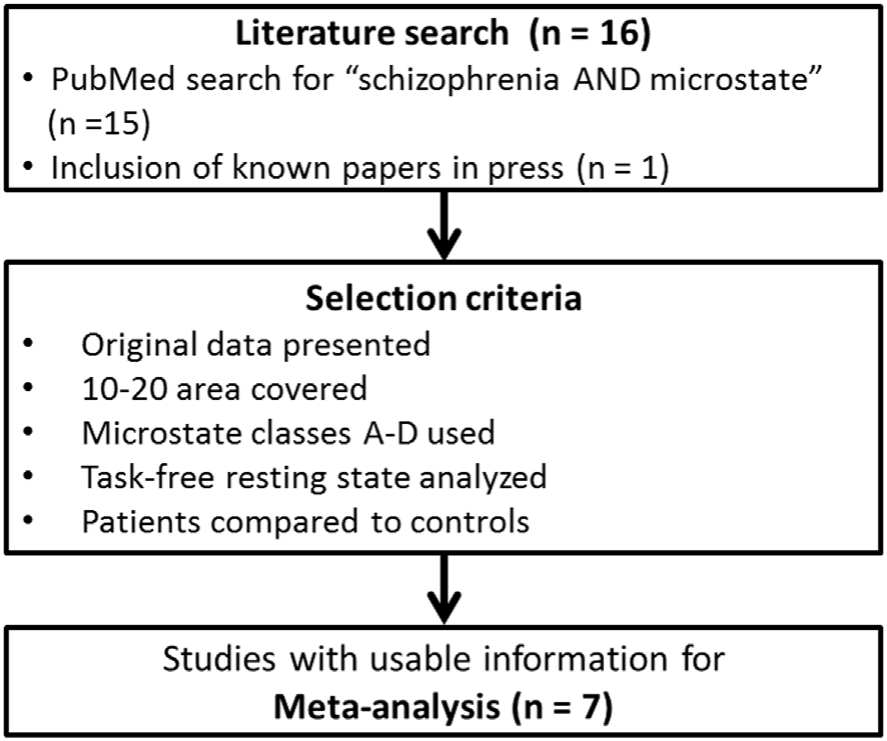
**Selection process of the included studies in the meta-analysis**.

To identify the relevant literature, we conducted a PubMed search with the terms “schizophrenia” and “microstate.” The resulting papers were then reviewed for particular criteria chosen to assure a sufficient comparability of the extracted microstate parameters across studies. These criteria were the following: (1) the paper reported original findings, (2) the electrode array used for the analysis covered at least the area of the standard 10-20 system, (3) four microstate classes were fitted to the data (being the most frequently used number of microstate classes across studies), and (4) subjects were recorded in a task-free resting-state condition. Care was taken to exclude the duplicate reporting of data in different papers. Note that the justification of the choice of these criteria was purely pragmatic; it identified the broadest common ground to compare studies on microstates in schizophrenia at the time of this paper being written. We do not imply that these criteria are the optimal choice to investigate microstates in general. In addition of the papers identified by the PubMed search, we were aware of, and included one more study that met the above criteria and had been accepted for publication, but was not yet registered in PubMed (28). The employed search and selection criteria are shown in Figure 1. A complete list of all papers found in PubMed with a brief rationale for inclusion/exclusion is given in Table S1 in Supplementary Material.

There was a total of seven studies meeting our criteria, beginning with a paper in 1999 (23) and ending with the most recent paper by Tomescu et al. (28). The included papers are shown in Table 1, together with the central characteristics of the studied population. Note, however, that the multicenter-study by Lehmann et al. (21) included a small (six patients and six controls) subset of subjects where the midline electrodes were interpolated. Since the data were only presented across all study centers and since there were many more subjects from other centers, we decided to include this study despite this fact.

**Table 1:** **Studies included**.

Reference	*n*	Diagnosis	Comorbidity included	Medication	Age	Gender		Channels	Recruitment area
						M	F		
Koenig et al. (23)	9	DSM-IV: 295.30 paranoid type	n.a.^a^	No	24.82 (range: ± 6.67)	3	6	19	Switzerland
		DSM-IV: 295.40 schizophrenieform dis.							
Lehmann et al. (21)	27	DSM-IV: 295.30 paranoid type	n.a.^a^	No	23.9 (SD: 4.5)	18	9	16–21^b^	Japan, Italy, and Germany
		DSM-IV: 295.90 undifferentiated type							
		DSM-IV: 295.10 hebephrenic type							
		DSM-IV: 295.20 catatonic type							
Kikuchi et al. (13)	21	DSM-IV: 295.30 paranoid type	n.a.	No	28.1	11	10	18	Japan
		DSM-IV: 295.10 disorganized type							
Nishida et al. (33)	18	DSM-IV: 295^c^	n.a.^a^	No	24.50 (SD: 6.3)	10	10	19	Japan
Andreou et al. (26)	18	DSM-IV: 295^c^	Depressive dis.	Yes	23.67 (SD: 4.4)	16	2	64	Germany
			Substance related dis.							
		Personality dis.						
Tomescu et al. (27)	30	High risk patients	Anxiety dis.	Yes	16.5 (range: ± 2.5)	13	17	204	Switzerland
			ADHD						
			Mood dis.							
		Schizophreniform dis.						
Tomescu et al. (28)	27^d^	DSM-IV: 295^c^	n.a.	Yes	34.5 (range: ± 9.5)	14	13	64	Georgia

*^a^Excluded, if it might involve or affect brain function*.

*^b^16: Italy, 19: Japan, and 21: Germany*.

*^c^No specification*.

*^d^Only part of the sample included to avoid overlapping samples*.

Importantly, all of these studies were based on a topographically consistent set of four prototypical microstate classes that were labeled from A to D according to the similarity of the obtained microstate classes with the microstate prototype maps reported in the first study of this kind (23). In addition, these papers were coherent and largely complete in the microstate parameters they reported, namely, coverage (percent total analysis time covered by each microstate class), occurrence (number of microstates observed per second for each microstate class), and mean duration (average duration of microstates of a given class). From all of these studies, mean and standard deviation (SD) of patient and control data were available for most of the typical microstate parameters and thus collected for the meta-analysis. Where data before and after medication were available, the data obtained before medication were used. An overview of the methodological content of the included studies is illustrated in Table 1.

The means and SDs of each study were used to compute hedges-g and its SD as standardized measure of within-study effect size. This computation was conducted using the package “compute.es” (34) of the R-software (35). The obtained within-study effect sizes were then weighted by their inverse variance to obtain a weighted mean effect size across studies, and the standard error of these mean effect sizes was extracted. In order to test the significance of the obtained overall mean effect sizes, a *Z*-test was conducted, and the upper and lower confidence intervals were computed. This yielded a total of 12 tests, each one assessing a potential difference between patients and controls in one of the three microstate features and in one of the four microstate classes. Since the single-subject data were not available, it was not possible to extend the analysis to a multifactorial level that would assess the overall significance of group by microstate class interactions. In order to correct for potential false positives due to multiple testing, a Bonferroni correction was applied with a factor of 8. This factor was chosen because all studies reported four microstate classes, and all studies reported two independent features (occurrence and mean duration) per microstate class. The sometimes additionally reported coverage can be computed from occurrence and mean duration and was thus not considered as an additional independent test.

Significant overall mean effects were tested for potential confounding effects of medication by excluding studies that had investigated patients under medication. The results of EEG microstate features yielding significant mean effects were displayed using forest plots.

## Results

**Figure 2:**
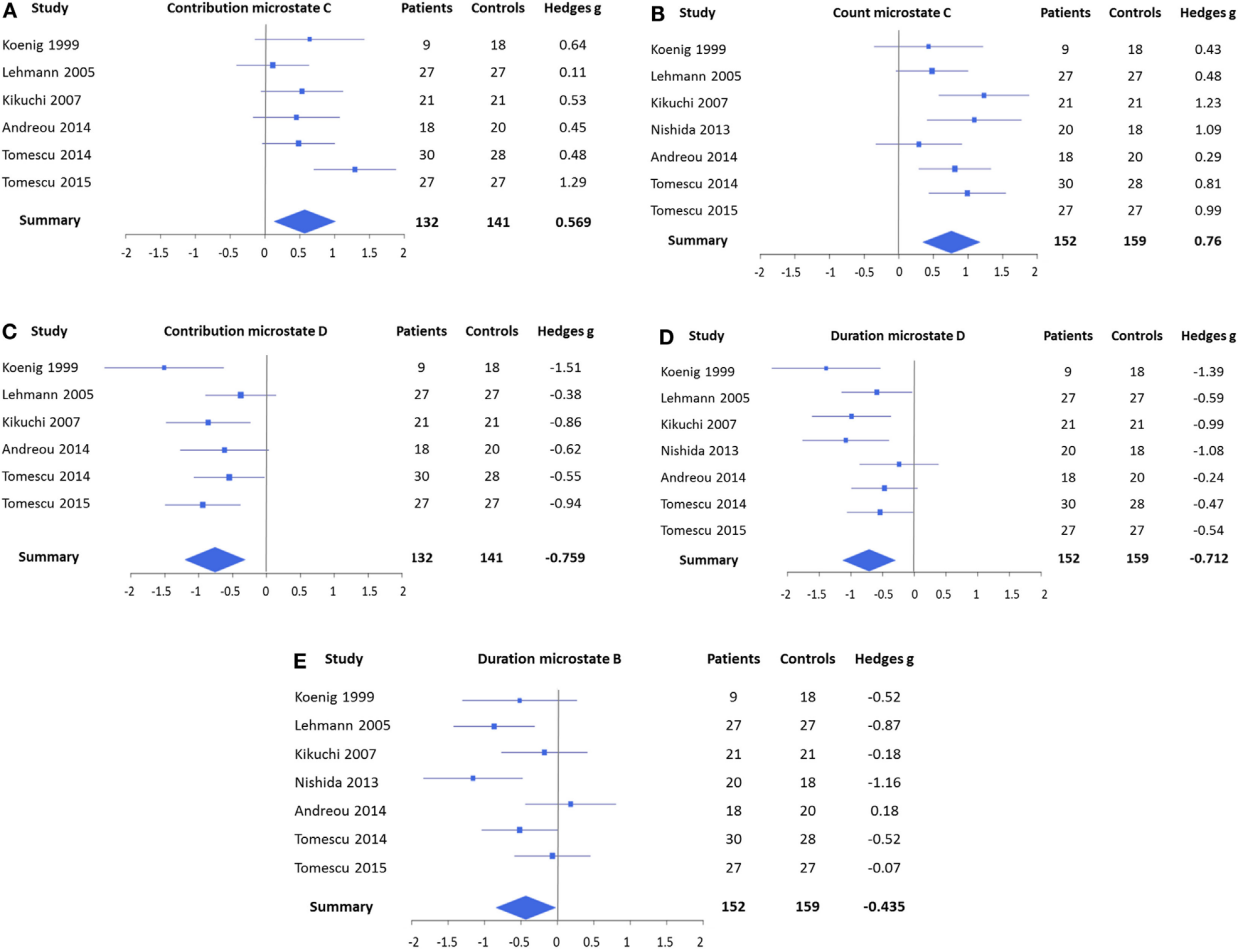
**Forest plots representing the EEG microstate features that yielded significant mean effects in the meta-analysis**. **(A)** Contribution of microstates of class C, which covered more percent time in patients, and **(B)** count of microstates of class C, which occurred also more frequently. **(C)** Contribution of microstates of class D, which covered less percent of time in patients, and **(D)** duration of microstates of class D, which was found to be shorter. **(E)** Duration of microstates of class B was shorter in patients. Note that the contribution of microstates of class C **(A)** and the duration of microstates of class B **(E)** were not significant after Bonferroni correction. No significant effects were found for mean duration of microstates of class C, count of microstates of class D, and count and contribution of microstates of class B.

The forest plots of the significant mean effect sizes are shown in Figure 2. The meta-analysis yielded significant mean effect sizes of medium size for microstates of class C, where a consistent increase was present and microstates of class D, where a reduction was observed. Namely, microstates of class C were found to occur more frequently in schizophrenia (*g* = 0.760, uncorrected *p* = 0.0003, and corrected *p* = 0.0024) and to cover more percent of time (*g* = 0.569, uncorrected *p* = 0.011, and corrected *p* = 0.088), whereas no significant effect was found for mean duration (*g* = 0.176 and uncorrected *p* = 0.39). Contrary to that, microstates of class D were found to cover less percent time (*g* = −0.759, uncorrected *p* = 0.0008, and corrected *p* = 0.0064) and to last shorter in the mean (*g* = −0.712, uncorrected *p* = 0.0007, and corrected *p* = 0.0056), whereas no consistent change in occurrence could be identified (*g* = −0.351 and uncorrected *p* = 0.090). When excluding studies with medicated patients, these effects were preserved and mostly showed increased effect sizes (contribution class C: *g* = 0.393, occurrence class C: *g* = 0.800, contribution class D: *g* = −0.821, and duration class D: *g* = −0.970), which makes it unlikely that these effects can be attributed to medication.

In addition, the meta-analysis showed a small, but significant, effect size for a shortening of microstate class B (*g* = −0.435 and *p* = 0.037) that also increased when only unmedicated patients were considered (*g* = −0.679 and *p* = 0.017). However, this effect was not significant after Bonferroni correction. The meta-analyses did not identify any consistent effects in microstate class A.

## Discussion

The present study introduces the overall evidence for alterations of EEG microstates in schizophrenia patients, based on all available studies that could reasonably be included in a meta-analysis. The inclusion criteria for the studies were defined by the pragmatic objective of maximizing the amount of studies to be compared, which implied that the included studies had quantified four microstate classes. The fact that the meta-analysis across those studies yielded evidence for consistent effects suggests that using four microstate classes has empirical justification and that these should be considered in future studies.

The main significant findings of the meta-analysis were related to microstates of classes C and D: microstates of class D were consistently found to cover less of the total time and showed a reduction in its mean duration, while microstates of class C covered more of the total time and occurred more frequently. There was also evidence for a weak effect in microstates of class B, where a shortening was observed. These effects could not reasonably be explained as side effects of medication upon the EEG, because they persisted when studies with medicated patients were excluded.

It has to be noted that our sample included one study with individuals at risk for developing schizophrenia [carrying the 22q11.2 deletion syndrome (27)]. This study showed the same microstate abnormalities in these individuals as in schizophrenia patients. This supports the view that microstate analysis can provide information of potential clinical value and could be considered useful for monitoring neuropsychiatric disorders.

There is an additional external support for the conclusion that in particular, microstate classes C and D relate to brain functions affected in schizophrenia patients: a large developmental study with healthy subjects found that during late adolescence, and thus during the typical age of onset of schizophrenia, microstate class D was found to be reduced compared to other life periods, while microstate C was most prominent in that phase of development (29). The above conclusion also receives support from interventional studies: Yoshimura et al. (36) found an increased duration in microstates of class D in healthy controls after a pharmacological intervention with a low dosage of an antipsychotic drug (perospirone) taken by healthy volunteers, indicating that the drug may counteract the microstate abnormalities observed in schizophrenia. In the study by Kikuchi et al. (13), EEG microstates were quantified in patients with schizophrenia before and after treatment with antipsychotic medication. In general, patients showed the above outlined pattern of increased C and decreased D microstate classes before treatment. When analyzing the microstates after treatment, this pattern had normalized selectively in those patients who responded well to the antipsychotic treatment, whereas patients with a poor response showed little change. In accordance with this findings, there was a strong negative correlation (*r* = −0.71) between the change of the symptoms and microstate class D duration, and a strong positive correlation between change of symptoms and microstate class C occurrence [*r* = 0.72 (13)].

Finally, in the domain of psychopathological symptoms, the paper by Koenig et al. (23) reported a negative correlation between microstates of class D duration and a score of paranoid-hallucinatory symptomatology, and Kindler et al. (37) could show that in schizophrenic patients with frequent auditory verbal hallucinations, shortening of microstate class D was associated with the acute experience of hallucinations.

### Comparison with Other Electrophysiological Indices of Schizophrenia

The effect sizes estimated in this meta-analysis of the currently available studies on EEG resting-state microstates lay between those found for spectral changes in resting-state EEG, and those found for amplitudes and latencies of various event-related potential (ERP) components: based on a total of over 1000 patients and controls each, a meta-analysis of spectral EEG indices indicated an effect size of 0.46 for an increase of delta band activity and an effect of 0.42 for an increase of theta band activity (38). Meta-analyses of ERP markers of schizophrenia showed larger effect sizes across rather different paradigms: P50 amplitude indices of sensory gating yielded effect sizes between −0.93 (39) and −1.56 (40), and the estimated effect size across studies using mismatch negativity type experiments was 0.99 (41). In studies that involved participants in active tasks, the effect size was estimated to be −0.83 for P300 abnormalities (42) and 0.82 for the N400 latency (43). Our intermediate effect size result may indicate that compared to frequency domain EEG indices, microstate analysis succeeds better in distinguishing between brain processes that are, or are not relevant for schizophrenia, but not to the same degree as some of the averaged evoked potentials do. However, by considering task-related activity as a state-dependent process, it follows that a particular type of resting-state abnormality may explain abnormal task responses in a broader range. Resting-state abnormalities may thus be causally “up-stream” of task-state abnormalities and hence become particularly interesting for an integral understanding of the observed psychopathology.

### What Is Known about the Function of the Affected Microstate Classes in Relation to Schizophrenia?

Microstates of class D have been attributed to flexible aspects of attention, such as switching and reorientation of attention to relevant information, because it has been associated with the frontoparietal attention network found in fMRI-BOLD data (19). Additionally, microstates of class D have been shown to be reduced in certain mental states, such as hypnosis (44), sleep (31), or during the acute phase of hallucinations (37). Noteworthy, all of these states are reality remote. This leads to the assumption that microstates of class D might be related to the updating of mental content based on internal and external information that is close to reality. The fact that our meta-analysis yielded evidence for an impairment of microstates of class D in patients with schizophrenia can thus be linked to deficits in attentional processes, context update, and executive control, where core deficits have long been identified in schizophrenia (45).

Microstate of class C, which, in contrast to microstates of class D, consistently occurred more often and covered more overall time in patients compared to healthy controls has been associated with the saliency network: areas found in fMRI studies that were associated with microstates of class C overlapped with the saliency network in the anterior cingulate, the inferior frontal gyrus, and the insula (19). Furthermore, an increase in microstates of class C has been found during hypnosis (44), which is a state often associated with the experience of saliency. Associating the increased occurrence of microstates of class C in schizophrenia with salience-related processes dovetails with the view that schizophrenia is a state of aberrant assignment of salience “at a mind level” (46).

The negative correlation between microstates classes C and D presence leads to the assumption of a balance between these states, with antagonistic functional roles: in conditions that demand an ongoing integration of contextual information, such as normal wakeful rest in healthy adults, microstates classes C and D explain an approximately equal part of the ongoing brain electric activity. However, microstates of class C become more dominant while ties to contextual information are loosened, i.e., during sleep, hypnosis, or psychosis, whereas microstates class D’s contribution is reduced. We may thus interpret the overall picture of our findings as an imbalance between attentional and saliency-related processes in schizophrenia patients. Interestingly, this observation is also supported by converging findings in two studies that analyzed the transitions between microstates (microstates syntax). We are particularly interested in their findings regarding microstates classes C and D in patients with schizophrenia or at risk for schizophrenia (28, 33): whereas healthy controls showed more than the expected transitions from C to D, patients showed these transitions less than expected, but there were more than expected transitions from D to C (28, 33). We may thus conclude that the research on microstates in schizophrenia yields a consistent picture of an imbalance between processes that integrate contextual information, which are reduced, and processes that load on saliency, which are increased.

Only little is known about microstates of class B. Britz et al. (19) related microstates of class B to the resting-state visual network in fMRI. In its role in schizophrenia patients, however, the results so far are inconsistent: two papers (21, 33) found a significant shorter duration, whereas one study (26) has reported significant more coverage of microstates of class B in patients compared to healthy controls, which would counteract an effect of shortening. In addition, the fact that the findings in microstate where not significant after correcting for multiple testing casts further doubt on the relevance of this effect.

### Implications and Future Directions

Cognition is subject to adaptive changes based on the external and internal needs. Furthermore, both cognitive resources and brain microstates are age-dependent and presumably undergo experience-dependent plastic changes (29). This may imply that microstates can be influenced by mental training such as NFB. Previously published studies have repeatedly shown that it is feasible to modulate stimulus-related EEG brain potentials with NFB in patient populations (47–49). In schizophrenia, Schneider et al. (50) found that patients were able to have conscious control of slow cortical potentials but no clinical changes were reported. Similarly, Gruzelier et al. (51) showed the capability of schizophrenic patients in learning a control task and demonstrated the feasibility of operant conditioning based on the EEG. Recently, one case series using EEG (52) and one study on resting-state fMRI (53) were published on NFB with schizophrenia patients. Both studies demonstrated learning.

The first indication of the ability of self-regulation of microstates of class D in a NFB-training in healthy controls has been shown by Diaz Hernandez et al. (32). All of the 20 trained subjects increased the percentage of time spent producing microstate D in at least one of the investigated NFB success indices. However, it remains to be seen if such a NFB training is also feasible in patients with schizophrenia and whether such a training would have a clinical effect.

The present study gave an overview of the few existing studies on brain microstates in schizophrenia that have accumulated over the last 15 years. Given the small number of studies that has been done so far, an enlargement of the sample size in future studies is essential. Furthermore, the heterogeneity of the included subjects in terms of medication and diagnosis gives reason to a cautious interpretation of the presented results. Nevertheless, despite small samples and heterogeneous subjects, we found consisting results with the included studies, which justifies further investigation of microstate classes C and D as state markers of acute schizophrenia and the attempt to modulate them (e.g., with help of NFB) as a possible add-on treatment.

## Author Contributions

KR, LDH, AB, and TK: substantial contributions to the conception or design of the work; or the acquisition, analysis, or interpretation of data for the work; drafting the work or revising it critically for important intellectual content; final approval of the version to be published; agreement to be accountable for all aspects of the work in ensuring that questions related to the accuracy or integrity of any part of the work are appropriately investigated and resolved.

## Conflict of Interest Statement

The authors declare that the research was conducted in the absence of any commercial or financial relationships that could be construed as a potential conflict of interest.
