# Supplementary material for: 15 Years of Microstate Research in Schizophrenia – Where Are We? A Meta-Analysis
Source: Front Psychiatry. 2016 Feb 26;7:22. doi: 10.3389/fpsyt.2016.00022 (PMC4767900; doi:10.3389/fpsyt.2016.00022)
Supplement: Supplementary file 1 [file table_1.docx]

|  | Author and Year | Title | Journal | Inclusion in meta-analysis |
| --- | --- | --- | --- | --- |
| 1 | Khanna et al., 2015 | *EEG: current status and future directions.* | Neuroscience & Biobehavioral Reviews | Excluded: Review paper |
| 2 | Tomescu et al., 2015 | *Schizophrenia patients and 22q11.2 deletion syndrome adolescents at risk express the same deviant patterns of resting state EEG microstates: A candidate endophenotype of schizophrenia* | Schizophrenia Research: Cognition | **Included** |
| 3 | Tomescu et al., 2014 | *Deviant dynamics of EEG resting state pattern in 22q11.2 deletion syndrome adolescents: A vulnerability marker of schizophrenia?* | Schizophrenia Research | **Included** |
| 4 | Andreou et al., 2014 | *Resting-state connectivity in the prodromal phase of schizophrenia: insights from EEG microstates.* | Schizophrenia Research | **Included** |
| 5 | Nishida et al., 2013 | *EEG microstates associated with salience and frontoparietal networks in frontotemporal dementia, schizophrenia and Alzheimer's disease* | Clinical Neurophysiology | **Included** |
| 6 | Schlegel et al., 2012 | *EEG microstates during resting represent personality differences* | Brain Topography | Excluded: only healthy subjects |
| 7 | Kindler et al., 2011 | *Resting-state EEG in schizophrenia: auditory verbal hallucinations are related to shortening of specific microstates* | Clinical Neurophysiology | Excluded: no control group |
| 8 | Begré et al., 2008 | *White matter anisotropy related to electrophysiology of first episode schizophrenia during NoGo inhibition* | Neurobiology of Disease | Excluded: subjects recording during task and not at rest |
| 9 | Kleinlogel et al., 2007 | *Increased NoGo-anteriorisation in first-episode schizophrenia patients during Continuous Performance Test* | Clinical Neurophysiology | Excluded: subjects recording during task and not at rest |
| 10 | Kikuchi et al., 2007 | *Native EEG and treatment effects in neuroleptic-naïve schizophrenic patients: time and frequency domain approaches* | Schizophrenia Research | **Included** |
| 11 | Irisawa et al., 2006 | *Increased omega complexity and decreased microstate duration in nonmedicated schizophrenic patients* | Neuropsychobiology | Excluded: only 3 microstates |
| 12 | Lehmann et al., 2005 | *EEG microstate duration and syntax in acute, medication-naive, first-episode schizophrenia: a multi-center study* | Psychiatry Research: Neuroimaging | **Included** |
| 13 | Strelets et al., 2003 | *Chronic schizophrenics with positive symptomatology have shortened EEG microstate durations* | Clinical Neurophysiology | Excluded: insufficient electrodes |
| 14 | Koenig et al., 1999 | *A deviant EEG brain microstate in acute, neuroleptic-naive schizophrenics at rest* | European Archives of Psychiatry and Clinical Neuroscience | **Included** |
| 15 | Stevens et al., 1997 | *Increased duration and altered topography of EEG microstates during cognitive tasks in chronic schizophrenia* | Psychiatry Research | Excluded: subjects recording during task and not at rest |
| 16 | Kochi et al., 1996 | *Event-related potential P300 microstate topography during visual one- and two-dimensional tasks in chronic schizophrenics* | European Archives of Psychiatry and Clinical Neuroscience | Excluded: subjects recording during task and not at rest |

Appendix Table 1.

List of all the studies searched for inclusion in the meta-analysis and its inclusion or exclusion based on the selection criteria.
